# Quackery in the Profession

**Published:** 1866-12

**Authors:** 


### Quackery in the Profession.

There are many mean practices resorted to by the genus quack to obtain practice, at the expense of the honest, straight-forward, unassuming, modest, professional gentleman. There are a hundred little sneakish tricks, which are never exposed, because practiced in the dark, and which some professional men may be guilty of, and yet retain a certain status of respectability in the profession, particularly if they be successful in their sordid aims.

But of all mean practices, of all gross insults to professional decency and honor, the practice of puffing in newspapers, of allowing one's name to be inserted in the local colums of a paper, by a friendly "penny-a-liner"—in connection with some "interesting case," or "great operation," or "successful operation," or "shocking accident," is about the meanest. It is a direct violation of the code of ethics.

This practice, we are sorry to say, is but too common, and not only winked at by many men who assume professional respectability, but by some, courted.

Witness the following item which appeared a few days ago in the columns of a daily paper in a neighboring city:

"Successful Surgical Operation.—Dr. ———, of this city, assisted by Dr. ———, and Dr. ———, successfully removed an ovarian tumor from the abdomen of a lady in this city yesterday afternoon, which weighed fully forty pounds. The operation was witnessed by a number of eminent medical gentlemen. We learn that the patient is doing finely this morning, and that the highest hopes are entertained of her ultimate recovery."

Charity obliges us to suppress the name of the individual in whose favor this "puff" appears, which bears intrinsic evidence of his having furnished the data upon which it is based, if not of having penned it himself. We quote this only as an instance of many, with a strong appeal to the profession to frown down this contemptible tendency to quackery in the profession.

People who are interested in any particular case of medical or surgical disease among their friends, will know and hear all about it without the case being paraded in a paper. What, then, is the object of such items? To "puff" the Doctor. There is a very simple remedy to stop such practices. Let respectable medical societies, by resolution, request the newspapers of their respective districts or cities to omit mentioning the name of any member in connection with any operation, case of disease, accident, etc. We venture to say that editors of newspapers will gladly comply with the request, and neither the public nor the profession will thereby be a loser. But it will force those addicted to the practice in the profession, either to stop it, or to put themselves outside the pale of professional respectability, where they really belong.—*Medical and Surgical Reporter.*

